# Exercise-induced abnormal recovery of heart rate and ventricular repolarization parameters among smokers without known cardiovascular disease: A cross-sectional study

**DOI:** 10.18332/tid/216383

**Published:** 2026-03-19

**Authors:** Ayhan Cosgun, Huseyin Oren

**Affiliations:** 1 Cardiology Department, Sincan State Hospital, Ankara, Turkey; 2 Department of Cardiology, Cihanbeyli State Hospital, Konya, Turkey; 3 Department of Cardiology, Ankara Bilkent City Hospital, Ankara, Turkey

**Keywords:** Tp-e interval, QT interval, Tp-e/QTc ratio, smoking, treadmill exercise testing

## Abstract

**INTRODUCTION:**

Smoking is a major preventable risk factor for cardiovascular disease and is strongly associated with sudden cardiac death (SCD). This study investigated the relationship between the heart rate recovery index (HRR-I), T peak–end recovery index (Tp-eR-I), and QT interval recovery index (QTR-I) in smokers compared with non-smokers.

**METHODS:**

This cross-sectional study, conducted in Bilkent City Hospital, Ankara, Turkey, between May 2017 and June 2025, included 150 healthy smokers (120 males, 30 females) and 123 healthy non-smokers (97 males, 26 females). Smoking data are self-reported. All participants underwent symptom-limited treadmill exercise testing using the Bruce protocol. Heart rate (HR), QT, and Tp-e intervals were measured at baseline, peak exercise, and during recovery periods. HRR-I was calculated as the difference between peak HR and HR at the 1st, 2nd, and 3rd minutes of recovery. Tp-eR-I and QTR-I were calculated as the differences between baseline values and those obtained during peak, 1st, 2nd and 3rd minutes recovery times.

**RESULTS:**

Smokers exhibited significantly lower HRR-I values at the 1st [18.6 ± 7.1 vs 24.2 ± 6.9, p<0.001; 17.9 (95% CI: 12.9–22.9)], 2nd [27.4 ± 7.3 vs 33.1 ± 7.1, p<0.001; 10.7 (95% CI 7.3–14.1)], and 3rd minutes [33.8 ± 7.9 vs 39.5 ± 8.3, p<0.001; 13.4 (95% CI: 9.6–17.2)] of recovery compared with non-smokers. Tp-eR-I and QTR-I values were significantly higher in smokers [Tp-eR-I: 7.8 ± 2.6 vs 5.2 ± 2.1, p<0.001; 18 (95% CI: 12–24)] [QTR-I: 24.5 ± 6.3 vs 18.9 ± 5.8, p<0.001; 12 (95% CI: 6–18)]. Smoking intensity was positively associated with Tp-eR-I (r=0.41, p<0.001) and QTR-I (r=0.36, p<0.001), and negatively associated with HRR-I (r= -0.39, p<0.001).

**CONCLUSIONS:**

Cigarette smoking impairs autonomic regulation as reflected by reduced HRR-I and abnormal ventricular repolarization recovery, shown by increased Tp-eR-I and QTR-I. These findings suggest that smoking disrupts sympathetic–parasympathetic balance and myocardial repolarization, potentially explaining the higher incidence of arrhythmias and sudden cardiac death among smokers.

## INTRODUCTION

Cigarette smoking remains one of the most important preventable causes of morbidity and mortality worldwide. The deleterious effects of smoking on the cardiovascular system have been well documented, including endothelial dysfunction, increased oxidative stress, autonomic imbalance, and electrophysiological alterations that predispose to arrhythmias and sudden cardiac death (SCD)^1-3^. Epidemiological data indicate that smokers have a two- to four-fold higher risk of developing coronary artery disease and SCD, compared with non-smokers^4,5^.

Electrocardiographic parameters have been widely used to evaluate the impact of smoking on cardiac electrophysiology. Among them, the QT interval and the T peak–end (Tp-e) interval are of particular importance as markers of ventricular repolarization and arrhythmogenic potential^6,7^. Prolongation of the QT interval has been associated with an increased risk of ventricular tachyarrhythmias and SCD, whereas Tp-e is considered a reliable index of transmural dispersion of repolarization^8-10^. Furthermore, Tp-e/QT and Tp-e/QTc ratios have been proposed as more stable indicators of ventricular repolarization heterogeneity^11^.

The treadmill exercise test (TET) provides additional information on the dynamic behavior of these parameters under sympathetic stimulation and recovery^12^. The heart rate recovery index (HRR-I), calculated as the difference between peak heart rate during exercise and heart rate at the first minute of recovery, reflects vagal reactivation and has been identified as a strong predictor of all-cause and cardiovascular mortality^13-15^. Recent studies suggest that impaired HRR is frequently observed in smokers, indicating autonomic dysfunction and increased arrhythmic susceptibility^16,17^.

However, data on the relationship between recovery indices of QT and Tp-e intervals (QTR-I and Tp-eR-I) and HRR-I in smokers are limited. Therefore, the present study aimed to investigate the interactions between HRR-I, QTR-I, and Tp-eR-I parameters in smokers and to explore their potential role in predicting the risk of malignant ventricular arrhythmia.

The aim of this study was to determine the effect of smoking on Tp-e and QT interval, Tp-e/QT, Tp-e/QTc ratios and HRR-I, and how these parameters changed with the exercise test.

## METHODS

### Study design and population

This cross-sectional study enrolled 150 smokers without cardiovascular disease (120 males and 30 females) who presented to the cardiology outpatient clinic with atypical chest pain. The study was conducted between May 2017 and June 2025 at Bilkent City Hospital, Ankara, Turkey. All participants had normal findings on clinical history, physical examination, and routine laboratory tests. A non-smokers group consisting of 123 healthy individuals (non-smoker) (97 males and 26 females), also free of disease according to history, physical examination, and laboratory results, was similarly recruited.

Exclusion criteria comprised systemic diseases such as diabetes mellitus or hypertension; abnormal hepatic or renal function tests; structural or significant valvular heart disease; diastolic dysfunction; electrolyte imbalance; anemia; thyroid dysfunction; documented coronary artery disease; permanent pacemaker implantation; current cardiac medication; tricuspid regurgitation greater than mild severity; cardiac chamber dilatation; chronic obstructive pulmonary disease or ongoing bronchodilator therapy; inability or unwillingness to perform pulmonary function testing; and a diagnosis of malignancy. Demographic characteristics, smoking history, and comorbidities were systematically recorded. After a five-minute rest period, all participants underwent a complete physical examination including three consecutive blood pressure measurements, followed by electrocardiography (ECG) and transthoracic echocardiography (TTE). Subjects with a mean blood pressure ≥140/90 mmHg were excluded.

### Ethics

All procedures involving human participants were conducted in accordance with the ethical standards of the relevant national research ethics committee and the 1964 Helsinki Declaration and its later amendments. Written authorization for the study was obtained from the Bilkent City Hospital Administration (Approval number: TABED 2-25-1603). Informed written consent was obtained from all participants before study enrollment.

### Electrocardiography

Standard 12-lead surface ECGs were obtained in the supine position using a commercially available ECG system (CardiofaxV model 9320, Nihon Kohden, Tokyo, Japan) at a paper speed of 25 mm/s and an amplitude of 10 mm/mV. Each 10-second tracing contained approximately 4–6 cardiac cycles per lead. ECG intervals were measured manually with a digital caliper (TorQ 150 mm Digital Caliper LCD) and a magnifying glass by a single cardiologist blinded to the smokers group. The QT and T peak-to-end (Tp-e) intervals were determined with a precision of 0.01 mm. Inter-rater reliability was assessed using the intraclass correlation coefficient (ICC), which was 0.81 for the first investigator (AC) and 0.77 for the second investigator (HO), indicating good measurement consistency.

### Exercise stress testing

Baseline heart rate, QT, and Tp-e intervals were recorded prior to exercise. Both groups then underwent treadmill exercise testing using the standard Bruce protocol^18^. Heart rate recovery (HRR) was measured at basal and peak state, 1st, 2nd and 3rd minutes post-exercise using (Cardiosis, TEPA), following standard protocols. QT and Tp-e intervals were measured at peak exercise and during the 1st, 2nd, and 3rd minutes of recovery, with simultaneous heart rate recordings. Corrected QT (QTc) and corrected Tp-e (cTp-e) intervals were calculated using Bazett’s formula, and Tp-e/QT as well as Tp-e/QTc ratios were derived^18^. Recovery indices for heart rate, QT, and Tp-e intervals were calculated by subtracting recovery phase values at the 1st, 2nd, and 3rd minutes from their respective peak exercise values.

### QT interval measurement

The QT interval was defined from the onset of the QRS complex to the return of the T wave to the isoelectric baseline. Measurements were performed in at least nine precordial leads and one limb lead, using three consecutive QRS complexes. The arithmetic mean of the measurements was used, and QTc values were calculated according to Bazett’s correction^18^.

### T peak-to-end interval measurement

The Tp-e interval was measured from the apex of the T wave to the point of return to the isoelectric line, primarily in leads V^2-5^. The raw values were multiplied by 40 to yield the interval in milliseconds. In the presence of a U wave, the end of the T wave was defined as the nadir between the T and U waves. Corrected Tp-e intervals were also calculated using Bazett’s formula^18^. Arithmetic means of multiple measurements were used for subsequent analysis.

### Statistical analysis

All statistical analyses were performed using SPSS software (version 26, IBM Corp., Armonk, NY, USA). Continuous variables were expressed as mean ± standard deviation (SD), and categorical variables as frequencies and percentages. The normality of parameters distribution was assessed using the Kolmogorov–Smirnov test. Non-normally distributed variables were expressed as median (interquartile range, IQR). Comparisons between smokers and non-smokers were performed using the independent samples Student’s t-test for normally distributed continuous variables and the Mann–Whitney U test for non-normally distributed variables. Categorical variables were compared using the chi-squared test. Repeated measures within groups (baseline, peak exercise, and recovery values) were analyzed using one-way repeated measures analysis of variance (ANOVA), followed by Tukey’s *post hoc* test for pairwise comparisons. It should be noted that repeated measures ANOVA was performed without adjustment for potential confounders such as age, sex, and BMI. This approach was chosen for simplicity in initial group comparisons, and we acknowledge that future analyses using multivariable models could further refine the assessment of the independent effects of smoking on recovery indices. Association between smoking parameters (cigarettes per day, duration of smoking, pack-years) and recovery indices (HRR-I, Tp-eR-I, QTR-I) were assessed using the Pearson correlation coefficient. A two-tailed p<0.05 was considered statistically significant.

### Power analysis

A power analysis was conducted using G*Power 3.1 to determine the adequacy of the sample size for detecting differences between smokers and non-smokers in the studied indices. Based on the observed means and pooled standard deviations, Cohen’s d effect sizes were calculated for each parameter: HRR-I (d=0.42), Tp-eR-I (d=0.65), and QTR-I (d=0.63). With a two-tailed α of 0.05 and a desired power of 0.8, the minimum required sample sizes per group were estimated as 46, 28, and 30, respectively. The actual sample sizes in this study (smokers: n=150; non-smokers: n=123) exceeded these requirements, indicating that the study was sufficiently powered to detect significant differences between groups.

### Data collection and variables

Comprehensive demographic and clinical data were systematically collected for all participants, including age, sex, and body mass index (BMI). Height and weight were measured using standardized procedures with a calibrated stadiometer and scale, and BMI was subsequently calculated as weight in kilograms divided by height in meters squared (kg/m^2^). Smoking status was assessed via structured self-report questionnaires, capturing both the daily number of cigarettes and the cumulative duration of smoking, to ensure accurate classification of participants as smokers or non-smokers. Electrocardiographic parameters, including Tp-e interval, QT interval, corrected QT (QTc), heart rate (HR), corrected Tp-e (cTp-e), and their respective ratios (Tp-e/QT, Tp-e/QTc), were obtained from standard 12-lead ECG recordings at baseline, at peak exercise during the treadmill exercise test (TET), and at the 1st, 2nd, and 3rd minutes of post-exercise recovery. Measurement procedures were standardized across all participants to minimize intra- and inter-observer variability. Potential confounding variables, including age, sex, and BMI, were identified *a priori* and considered for adjustment in multivariable regression models to rigorously evaluate their potential influence on the observed electrocardiographic outcomes, thereby enhancing the robustness and interpretability of the study findings.

## RESULTS

The mean smoking exposure in the smokers group was 17.5 ± 9.2 pack-years. The parameters on smoking behavior are based on participants’ self-reporting; no biochemical verification was performed. The mean age of the smokers group was 56.74 ± 12.7 years, whereas the mean age in the non-smokers group was 57.35 ± 12.4 years.

When the sociodemographic and baseline clinical characteristics of the study and non-smokers groups were compared, there were no statistically significant differences in terms of age, gender distribution, body mass index (BMI), blood pressure, left ventricular mass, glucose, thyroid stimulating hormone (TSH), lipid profile, electrolytes, calcium, magnesium, or creatinine levels (all p>0.05). However, the basal heart rate was significantly higher in the smoker group compared to the non-smoker group (79.3 ± 9.8 vs 69.7 ± 10.1 beats/min, p<0.0001) (Table 1).

**Table 1:** Sociodemographic and baseline clinical characteristics of smokers (N=150) and non-smokers (N=123), at Bilkent City Hospital, Ankara, Turkey, 2017–2025

*Variable*	*Smokers (N=150) Mean ± SD*	*Non-smokers (N=123) Mean ± SD*	*T/Z*	*p*	*Mean difference (95% CI)*
**Age** (years)	56.7 ± 12.7	57.4 ± 12.5	0.24	0.80	-0.7 (-3.69–2.29)
Male (%)	80.0	78.8	Z=0.02	0.88	
Female (%)	20.0	21.2	Z=0.02	0.88	
Body mass index (kg/m²)	24.7 ± 2.2	25.1 ± 2.3	1.0	0.31	-0.4 (-0.94–0.14)
Basal heart rate (beats/min)	79.3 ± 9.8	69.7 ± 10.1	-7.94	<0.01	9.6 (7.22–11.98)
Systolic blood pressure (mmHg)	122.6 ± 11.5	119.4 ± 10.6	1.5	0.13	3.2 (0.57–5.83)
Left ventricular mass (g)	178.2 ± 22.1	175.5 ± 19.5	0.67	0.50	2.7 (-2.24–7.64)
Glucose (mg/dL)	95.5 ± 9.5	94.2 ± 11.7	0.57	0.56	1.3 (-1.27–3.87)
Thyroid stimulating hormone (mU/L)	2.2 ± 0.56	2.31 ± 0.4	0.89	0.37	-0.11 (-0.22–0.0)
Total cholesterol (mg/dL)	178.0 ± 25.5	173.6 ± 30.5	0.78	0.43	4.4 (-2.36–11.16)
Triglycerides (mg/dL)	124.7 ± 13.7	122.6 ± 10.6	0.89	0.37	2.1 (-0.78–4.98)
Low-density lipoprotein (mg/dL)	136.4 ± 22.6	132.8 ± 22.6	0.80	0.42	3.6 (-1.79–8.99)
Sodium (mEq/L)	142.5 ± 1.6	142.4 ± 1.4	0.36	0.71	0.1 (-0.26–0.46)
Calcium (mg/dL)	9.6 ± 0.3	9.7 ± 0.5	1.09	0.47	-0.1 (-0.2–0.0)
Potassium (mEq/L)	3.8 ± 0.2	3.9 ± 0.3	1.87	0.064	-0.1 (-0.16 – -0.04)
Magnesium (mg/dL)	2.18 ± 0.1	2.2 ± 0.1	1.20	0.23	-0.02 (-0.04–0.0)
Creatinine (mg/dL)	0.95 ± 0.1	0.93 ± 0.1	1.54	0.12	0.02 (-0.0–0.04)

The normality of parameters distribution was assessed using the Kolmogorov–Smirnov test. Non-normally distributed data were compared using the Mann–Whitney U test.

Electrocardiographic findings revealed that basal Tp-e interval, QT interval, and heart rate, as well as peak, 1st minute, 2nd minute, and 3rd minute Tp-e, QT, and heart rate values during treadmill exercise testing were significantly higher in the smokers group compared with the non-smokers group (all p<0.01) (Table 2).

**Table 2:** Comparison of electrocardiographic findings between smokers (N=150) and non-smokers (N=123) at Bilkent City Hospital, Ankara, Turkey, 2017–2025

*Variable*	*Smokers Mean ± SD*	*Non-smokers Mean ± SD*	*T*	*p*	*Mean difference (95% CI)*
Tp-e basal	79.3 ± 9.8	69.7 ± 10.1	7.94	<0.01	9.6 (7.5–11.7)
QT basal	347.3 ± 19.9	321.4 ± 30.4	8.13	<0.01	25.9 (18.4–33.4)
HR basal	91.6 ± 14.1	85.3 ± 11.3	4.61	<0.01	6.3 (3.4–9.2)
Tp-e peak	54.2 ± 7.8	43.6 ± 7.2	11.6	<0.01	10.6 (8.8–12.4)
QT peak	251.5 ± 13.3	237.5 ± 18.3	7.3	<0.01	14.0 (10.3–17.7)
HR peak	165.7 ± 7.2	158.8 ± 8.7	7.2	<0.01	6.9 (4.9–8.9)
Tp-e 1st min	63.2 ± 9.6	48.6 ± 8.9	12.9	<0.01	14.6 (11.9–17.3)
QT 1st min	265.5 ± 19.6	247.6 ± 22.4	7.03	<0.01	17.9 (12.9–22.9)
HR 1st min	135.1 ± 14.6	124.0 ± 11.2	6.92	<0.01	11.1 (7.7–14.5)
Tp-e 2nd min	71.1 ± 8.5	53.3 ± 10.3	15.6	<0.01	17.8 (15.2–20.4)
QT 2nd min	291.8 ± 20.6	267.4 ± 28.7	8.1	<0.01	24.4 (18.5–30.3)
HR 2nd min	118.4 ± 14.5	107.7 ± 10.4	6.8	<0.01	10.7 (7.3–14.1)
Tp-e 3rd min	79.8 ± 11.8	58.2 ± 10.6	15.7	<0.01	21.6 (18.5–24.7)
QT 3rd min	311.5 ± 24.9	281.2 ± 28.4	9.3	<0.01	30.3 (23.5–37.1)
HR 3rd min	111.7 ± 16.7	98.3 ± 10.2	7.7	<0.01	13.4 (9.6–17.2)

All variables are continuous and showed a normal distribution; therefore, the Student’s t-test (parametric test) was used for comparisons between groups. A p<0.05 was considered statistically significant. Tp-e: T peak-to-end interval. QT: QT interval. HR: heart rate (beats per minute). cTp-e: corrected Tp-e interval. QTc: corrected QT interval. Tp-e/QT: Tp-e divided by QT interval. Tp-e/QTc: Tp-e divided by QTc interval.

**Figure 1:**
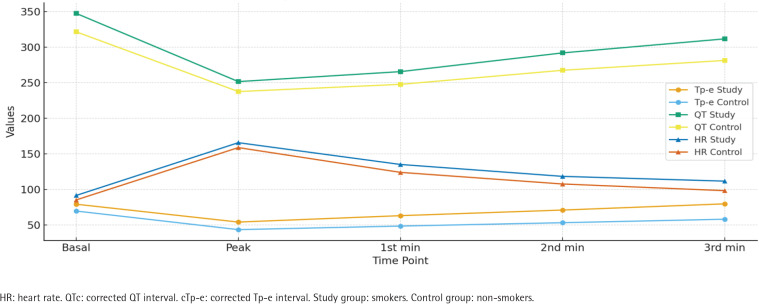
Electrocardiographic parameters of smokers (N=150) and non-smokers (N=123), at Bilkent City Hospital, Ankara, Turkey, 2017–2025

Regarding corrected values and ratios, the smokers group demonstrated significantly higher basal cTp-e, QTc, Tp-e/QT, and Tp-e/QTc values compared to the non-smokers group. Similarly, peak, 1st, 2nd, and 3rd minute corrected Tp-e, QTc, Tp-e/QT, and Tp-e/QTc values were all significantly higher in the smokers group (all p<0.01) (Table 3, Figure 1).

**Table 3:** Comparison of corrected values and ratios between the smokers (N=150) and non-smokers (N=123), at Bilkent City Hospital, Ankara, Turkey (2017–2025)

*Variable*	*Smoker Mean ± SD*	*Non-smoker Mean ± SD*	*Mean difference (95% CI)*	*T*	*p*
cTp-e basal	97.5 ± 13.0	83 ± 11.1	14.5 (10.8–18.2)	9.8	<0.01
QTc basal	429.4 ± 30.5	393.7 ± 23.8	35.7 (24.6–46.8)	4.1	<0.01
Tp-e/QT basal	0.25 ± 0.03	0.20 ± 0.03	0.05 (0.04–0.06)	13.7	<0.01
Tp-e/QTc basal	0.21 ± 0.02	0.17 ± 0.03	0.04 (0.03–0.05)	9.9	<0.01
cTp-e peak	90.5 ± 12.7	70.8 ± 10.7	19.7 (16.2–23.2)	13.3	<0.01
QTc peak	406.6 ± 26.8	386.35 ± 14.6	20.25 (14.1–26.4)	7.5	<0.01
Tp-e/QT peak	0.24 ± 0.03	0.18 ± 0.02	0.06 (0.05–0.07)	12.7	<0.01
Tp-e/QTc peak	0.18 ± 0.02	0.11 ± 0.02	0.07 (0.06–0.08)	12.3	<0.01
cTp-e 1st min	106.5 ± 13.7	92.7 ± 13.2	13.8 (10.1–17.5)	8.4	<0.01
QTc 1st min	405.7 ± 27.6	380.5 ± 17.9	25.2 (16.9–33.5)	4.7	<0.01
Tp-e/QT 1st min	0.27 ± 0.03	0.24 ± 0.03	0.03 (0.02–0.04)	8.2	<0.01
Tp-e/QTc 1st min	0.18 ± 0.02	0.16 ± 0.02	0.02 (0.01–0.03)	4.1	<0.01
cTp-e 2nd min	88.6 ± 13.9	78.5 ± 10.5	10.1 (6.8–13.4)	6.4	<0.01
QTc 2nd min	411.3 ± 32.4	383.2 ± 23.9	28.1 (19.5–36.7)	7.9	<0.01
Tp-e/QT 2nd min	0.27 ± 0.03	0.23 ± 0.03	0.04 (0.03–0.05)	11	<0.01
Tp-e/QTc 2nd min	0.20 ± 0.02	0.17 ± 0.03	0.03 (0.02–0.04)	9.9	<0.01
cTp-e 3rd min	103 ± 15.7	85.6 ± 12.7	17.4 (12.8–21.9)	9.9	<0.01
QTc 3rd min	418.4 ± 20.7	399 ± 24.4	19.4 (12.1–26.7)	7.1	<0.01
Tp-e/QT 3rd min	0.26 ± 0.04	0.21 ± 0.02	0.05 (0.04–0.06)	12.6	<0.01
Tp-e/QTc 3rd min	0.19 ± 0.03	0.17 ± 0.02	0.02 (0.014–0.026)	6.3	<0.01

All variables are continuous and showed a normal distribution; therefore, the student’s t-test (parametric test) was used for comparisons between groups. A p<0.05 was considered statistically significant. cTp-e: Corrected Tp-e interval. QTc: corrected QT interval.

Analysis of treadmill exercise testing (ANOVA) showed significant differences in both groups for Tp-e, QT, cTp-e, QTc, Tp-e/QT, Tp-e/QTc, and heart rate across the different stages of exercise (all p<0.01) (Table 4, Figure 3). The observed differences are presented without adjustment for potential confounders, which should be considered when interpreting these findings.

**Table 4:** Comparison of treadmill exercise test findings (ANOVA analysis) of smokers (N=150) and nonsmokers (N=123), at Bilkent City Hospital, Ankara, Turkey, 2017–2025

*Variable*	*Smokers*			*Non-Smokers*		
	*F*	*p*	*Mean difference (95% CI)*	*F*	*p*	*Mean difference (95% CI)*
Tp-e	66.5	<0.00001	32 (25–39)	34.1	<0.01	18 (12–24)
QT	147	<0.00001	40 (32–48)	140.8	<0.01	36 (28–44)
cTp-e	44.7	<0.00001	22 (16–28)	10.6	<0.01	8 (4–12)
QTc	2.67	0.034	6 (1–11)	6.4	<0.01	12 (6–18)
Tp-e/QT	25.7	<0.00001	0.05 (0.03–0.07)	21.9	<0.01	0.04 (0.02–0.06)
Tp-e/QTc	70.7	<0.00001	0.06 (0.04–0.08)	32.6	<0.01	0.03 (0.02–0.05)
HR	70.1	<0.00001	10 (7–13)	65.5	<0.01	9 (6–12)

These variables were continuous and met the assumption of normal distribution. For comparisons of within-group and between-group variances, the ANOVA test (parametric test) was applied. Statistical significance was defined as p<0.05. Repeated measures within groups (baseline, peak exercise, and recovery values) were analyzed using one-way repeated measures analysis of variance (ANOVA), followed by Tukey’s *post hoc* test for pairwise comparisons. HR: heart rate. QTc: corrected QT interval. cTp-e: corrected Tp-e interval.

**Figure 2:**
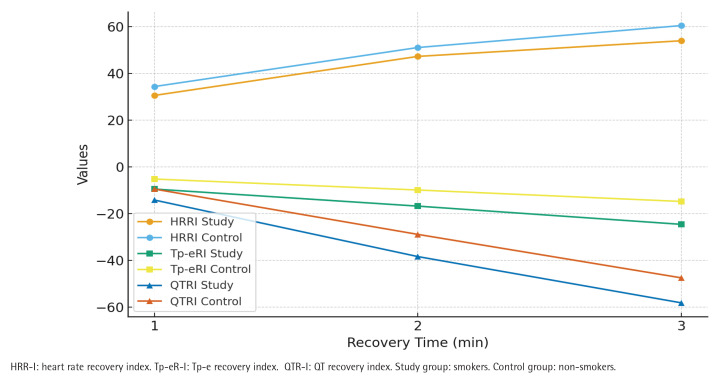
Recovery parameters of smokers (N=150) and non-smokers (N=123), at Bilkent City Hospital, Ankara, Turkey, 2017–2025

In terms of recovery indices, the smoker group exhibited significantly lower HR recovery indices (HRR-I_1_, HRR-I_2_, and HRR-I_3_) compared with the non-smokers group (p<0.01) (Figure 2). Furthermore, Tp-e recovery indices (Tp-eR-I_1_, Tp-eR-I_2_, Tp-eR-I_3_) and QT recovery indices (QTR-I_1_, QTR-I_2_, QTR-I_3_) were significantly more impaired in the smokers group compared with controls (p<0.05 for all).

**Figure 3:**
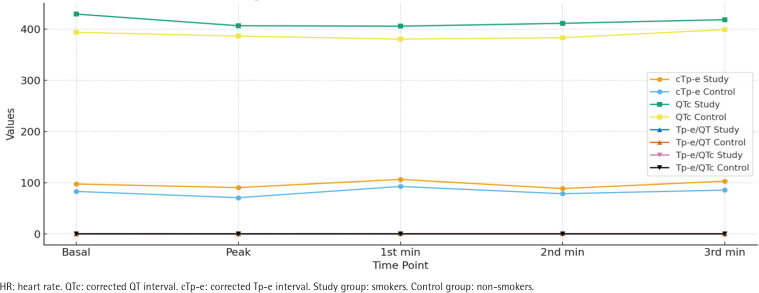
Corrected values of smokers (N=150) and non-smokers (N=123), at Bilkent City Hospital, Ankara, Turkey, 2017–2025

Correlation analysis demonstrated strong negative correlations between HR recovery indices and Tp-e recovery indices in both the study and non-smokers groups, with a more pronounced association in the smokers group (HRR-I_1_ vs Tp-eR-I_1_, r= -0.76, p<0.01). Additionally, moderate negative correlations were observed between HRR-I and QTR values, while Tp-eR-I and QTR indices were positively correlated in both groups. The strength of these associations, as assessed by Pearson correlation coefficients, was higher in the smokers group compared with the non-smokers group (Table 5).

**Table 5:** Comparison of recovery indexes of HR, Tp-e, and QT interval values of smokers (N=150) and nonsmokers (N=123), at Bilkent City Hospital, Ankara, Turkey, 2017–2025

*Variable*	*Smokers Mean ± SD*	*Non-smokers Mean ± SD*	*Mean difference (95% CI)*	*t*	*p*
HRR-I_1_	30.6 ± 11.7	34.4 ± 3.7	-3.80 (-6.91 – -0.69)	3.7	<0.01
HRR-I_2_	47.3 ± 13.4	51.1 ± 5.6	-3.80 (-7.47 – -0.13)	2.9	<0.01
HRR-I_3_	54.0 ± 17.2	60.5 ± 6.8	-6.50 (-11.18 – -1.82)	3.8	<0.01
Tp-eR-I_1_	-9.5 ± 18.2	-5.2 ± 13.4	-4.30 (-10.02–1.42)	2.1	0.03
Tp-eR-I_2_	-16.8 ± 15.7	-9.9 ± 8.7	-6.90 (-11.44 – -2.36)	6.4	<0.01
Tp-eR-I_3_	-24.6 ± 9.8	-14.8 ± 7.4	-9.80 (-12.43 – -7.17)	9.1	<0.01
QTR-I_1_	-14.2 ± 21.3	-9.5 ± 14.6	-4.70 (-10.85–1.45)	2.07	0.03
QTR-I_2_	-38.4 ± 19.6	-28.9 ± 17.8	-9.50 (-14.92 – -4.08)	4.1	<0.01
QTR-I_3_	-58.2 ± 21.1	-47.5 ± 16.5	-10.70 (-16.31 – -5.09)	4.5	<0.01

HRR-I: heart rate recovery index. Tp-eR-I: Tp-e recovery index. QTR-I: QT recovery index. 1: first minute recovery value. 2: second minute recovery value. 3: third minute recovery minute. The HRR-I, Tp-eR-I, and QTR-I indices are continuous variables. The normality of the parameters was assessed, and since most variables were normally distributed, the Student’s t-test was used. For variables that did not follow a normal distribution, the Mann–Whitney U test (non-parametric test) was applied. A p<0.05 was considered statistically significant. These variables were continuous and met the assumption of normal distribution. For comparisons of within-group and between-group variances, the ANOVA test (parametric test,) followed by Tukey’s *post hoc* test for pairwise comparisons, was applied.

## DISCUSSION

Our findings demonstrate a significant impairment in autonomic regulation among smokers, as evidenced by a pronounced reduction in heart rate recovery index (HRR-I) compared with non-smokers. These results are consistent with previous studies reporting adverse effects of cigarette smoking on autonomic cardiovascular function^19,20^, reinforcing the notion that smoking disrupts normal autonomic recovery following exertion. Moreover, while prior research has primarily focused on populations with existing cardiovascular risk factors, our study extends these observations to a cohort of otherwise healthy individuals, highlighting that even in the absence of overt disease, smoking exerts measurable deleterious effects on cardiac autonomic modulation. This comparison with the existing literature underscores both the robustness and the clinical relevance of our findings^12,21^.

Cigarette smoking profoundly disrupts the delicate balance of autonomic control, attenuating parasympathetic activity while simultaneously exaggerating sympathetic tone, thereby delaying vagal reactivation after exercise and perpetuating sustained sympathetic dominance^22^. This maladaptive autonomic response is not merely a transient physiological alteration but rather a persistent derangement that has been consistently identified as an independent predictor of adverse cardiovascular outcomes^23-25^, including increased morbidity and premature mortality^26,27^. Such findings underscore the central role of autonomic dysregulation in the cardiovascular toxicity of smoking.

In addition to autonomic impairment, our parameters revealed that smokers exhibited a striking prolongation of the Tp-e interval, accompanied by significantly elevated Tp-e/QT and Tp-e/QTc ratios^16,19,28^. These indices are not simply markers of altered electrophysiology but well-validated parameters that reflect increased heterogeneity of ventricular repolarization. The pathophysiological relevance of these disturbances lies in their established predictive value for malignant ventricular arrhythmias and sudden cardiac death (SCD)^11,13,14^. A growing body of literature has consistently demonstrated that chronic smoking promotes electrical remodeling by prolonging the Tp-e interval and augmenting Tp-e/QT ratios, thereby generating a pro-arrhythmogenic substrate that facilitates re-entrant circuits and malignant rhythm disturbances^2,16^. The current findings reinforce this mechanistic link, offering robust support for the hypothesis that smoking-induced ventricular repolarization abnormalities^29-31^ represent an important pathway connecting tobacco exposure to arrhythmic risk.

The coexistence of profound autonomic dysfunction and significant abnormalities in ventricular repolarization parameters among smokers provides a highly plausible mechanistic explanation for their markedly elevated risk of SCD. Beyond electrophysiological markers, large-scale epidemiological parameters further substantiate this association, demonstrating that cigarette smoking increases resting heart rate, attenuates the rise in heart rate during exercise, limits the achievement of maximal exercise capacity, and significantly delays post-exercise heart rate recovery^15,17^. Moreover, smoking is strongly associated with prolonged QT dispersion and elevated rate–pressure product, two additional markers of increased myocardial oxygen demand and electrical instability, which synergistically amplify the risk of malignant arrhythmias^11,32^.

Taken together, these findings indicate that cigarette smokers are subject to both structural and functional perturbations in cardiac electrophysiology and autonomic function. The simultaneous presence of prolonged Tp-e interval, elevated Tp-e/QT and Tp-e/QTc ratios, and markedly impaired HRR-I highlights the synergistic impact of autonomic dysregulation and ventricular electrical remodeling. This dual impairment constitutes a critical mechanistic axis through which smoking exerts its deleterious effects on arrhythmic vulnerability and the risk of SCD^5,10,11^. Considering the cross-sectional nature of this study and the reliance on self-reported smoking data, future prospective cohort studies are warranted to establish causal relationships and further elucidate the long-term effects of cigarette smoking on autonomic cardiac function.

### Limitations

This study has several limitations that should be acknowledged. Conducted at a single tertiary care center with a relatively small sample size, its findings may have limited external validity and might not be generalizable to broader populations or different ethnic, cultural, or healthcare settings. The cross-sectional nature of the study precludes any inference of causality; thus, the observed associations between smoking and repolarization abnormalities cannot establish temporal or causal relationships. Moreover, the potential for reverse causality cannot be completely excluded – individuals with subclinical cardiac changes might have altered smoking behavior due to early symptoms or health concerns.

Smoking exposure was based on self-reported data, which introduces potential information bias and misclassification error, as participants may have under- or over-reported their smoking status. Although key quantitative measures such as duration, intensity, and pack-years were recorded, other relevant factors – including nicotine dependence, type of tobacco product, and exposure to passive smoke – were not comprehensively evaluated. Residual confounding from unmeasured behavioral, environmental, or genetic variables – such as diet, alcohol consumption, physical activity, psychological stress, occupational exposures, or socioeconomic disparities – could have further influenced the findings.

Furthermore, the study did not incorporate advanced imaging modalities (e.g. cardiac MRI, echocardiographic strain analysis) or electrophysiological testing, which could provide deeper insights into structural or functional cardiac remodeling. Laboratory parameters reflecting oxidative stress, inflammation, or autonomic function were not assessed, limiting the mechanistic interpretation.

The relatively small sample size restricted the ability to perform subgroup analyses (e.g. sex-based or dose-response assessments) and interaction analyses (e.g. group × time effects). The lack of longitudinal follow-up for arrhythmic events or major cardiovascular outcomes prevents evaluation of prognostic significance. Finally, the study population consisted solely of individuals without known cardiovascular disease, which, while ensuring a homogenous baseline, limits applicability to populations with pre-existing cardiac conditions.

Future studies with larger, multi-center, and ethnically diverse cohorts, longitudinal designs, objective biomarkers of tobacco exposure, and comprehensive adjustment for confounders are warranted to confirm and expand upon these findings.

## CONCLUSIONS

Cigarette smoking is associated with impaired autonomic regulation, evidenced by reduced HRR-I, and with abnormal ventricular repolarization, demonstrated by prolonged Tp-e interval and elevated Tp-e/QT and Tp-e/QTc ratios. These alterations contribute to an increased arrhythmogenic substrate and may explain the elevated risk of malignant arrhythmias and sudden cardiac death among smokers^5,10,11,19,20,28^. The simultaneous evaluation of HRR-I, QTR-I, and Tp-eR-I during exercise testing may serve as a practical, non-invasive tool for identifying high-risk individuals, especially in populations with a high prevalence of smoking. Preventive strategies, including smoking cessation^33-35^, remain crucial to reducing the burden of cardiovascular morbidity and mortality^18,35,36^.

## Data Availability

The data supporting this research are available from the authors on reasonable request.
